# A qualitative study of interest in and preferences for potential medications to treat methamphetamine use disorder

**DOI:** 10.1186/s13722-023-00401-1

**Published:** 2023-08-16

**Authors:** Karla D. Wagner, Charles Marks, Phillip Fiuty, Robert W. Harding, Kimberly Page

**Affiliations:** 1 School of Public Health, University of Nevada, 1664 N. Virginia St. MC 0274, Reno, NV 89557 USA; 2 The Mountain Center Harm Reduction Center, 1000 North Paseo de Onate, Española, NM USA; 3 University of New Mexico Health Sciences Center, 1 University of New Mexico, MSC10 5550, Albuquerque, NM 87131-0001 USA

**Keywords:** Methamphetamine, Pharmacotherapies, Qualitative research, Patient-centered outcomes

## Abstract

**Introduction:**

We examined acceptability of and preferences for potential medications for treating methamphetamine use disorder (MUD) among people who use methamphetamine and examined how benefits and drawbacks of methamphetamine use affect perceived acceptability and preferences.

**Methods:**

We conducted qualitative interviews as part of a larger study in 2019–2020. The interview assessed patterns of substance use (including methamphetamine), benefits and drawbacks of methamphetamine use, and interest in a medication to treat MUD. Analysis used an inductive thematic approach, guided by three primary questions: (1) would participants be interested in taking a potential medication for MUD?; (2) what effects would they would like from such a medication?; and (3) what would their ideal treatment route and schedule be (e.g. daily pill, monthly injection)?.

**Results:**

We interviewed 20 people reporting methamphetamine use in the past 3 months (10 from Reno, Nevada, USA and 10 from Rio Arriba County, New Mexico, USA). Seven used exclusively methamphetamine, while thirteen used other substances in addition to methamphetamine. Most were enthusiastic about a potential medication to treat MUD. Of those who were not interested (n = 5), all indicated no current concerns about their methamphetamine use. Perceived functional benefits of methamphetamine use (i.e., energy, counteracting opioid sedation, and improved social and emotional wellbeing) informed preferences for a replacement-type medication that would confer the same benefits while mitigating drawbacks (e.g., psychosis, hallucinations, withdrawal). Opinions on preferred dosing varied, with some preferring longer acting medications for convenience, while others preferred daily dosing that would align with existing routines.

**Conclusion:**

Participants were excited about a potential for a medication to treat MUD. Their preferences were informed by the functional role of methamphetamine in their lives and a desire to maintain the stimulant effects while mitigating harms of illicit methamphetamine. Treatment outcomes that emphasize functioning and wellbeing, rather than abstinence, should be explored.

## Background

Methamphetamine use and associated health harms in the United States have increased significantly in recent years. The rate of overdoses associated with psychostimulants increased more than 3-fold from 2013 to 2019 [1]. Other prevalence indicators, including emergency department visits, calls to poison control centers, and drug seizures, also demonstrate increases across the US [2]. In 2019, there were an estimated 1,048,000 people in the US with methamphetamine use disorder, up from 684,000 to 2016 [3].

While the prevalence of methamphetamine use and MUD have increased [2], availability of evidence-based treatment lags. Contingency Management is currently the treatment strategy with the best evidence of effectiveness for MUD [4, 5], though concerns about cost and durability limit its scalability and uptake has been limited. There are currently no FDA-approved pharmacotherapies to treat (MUD; [6, 7]), though preliminary studies on some medications (i.e., mirtazapine, modafinil, bupropion, naltrexone, and agonist replacement medications) have demonstrated promising (but small) effects [8–11]. Basic science research to identify additional medication targets and strategies (including devices, vaccines, and monoclonal antibodies) is ongoing, but even the most promising candidates have not entered Phase III trials; medications remain at least 4–6 years away from patients in the clinical trial pipeline [12].

Currently the FDA requires a period of sustained abstinence be assessed as the primary outcome for trials of medications for treatment of substance use disorder, including for methamphetamine [13]. However, a focus on patient-reported outcomes such as functioning and health-related quality of life, rather than complete abstinence, may be more appropriate given the unique neurocognitive effects of long-term methamphetamine use and the high probability of relapse [14, 15]. Functional domains of interest include medical, employment, family/social, legal, and psychological wellbeing, as well as use of other drugs and alcohol. In fact, these outcomes have already been proposed in the context of treatment for opioid use disorder [16, 17]. In addition, there is evidence that reductions in methamphetamine use might confer cardiovascular benefits even in the absence of total abstinence [18].

To inform drug development, clinical trials research, and eventual service provision with a patient-centered perspective [19], the purpose of this study was to examine the acceptability of and preferences for a *potential* medication for treating MUD among a sample of people who use methamphetamine in Nevada and New Mexico, USA.

## Methods

### Setting

This study was conducted between December 2019 and February 2020 in Reno, Nevada, USA and Rio Arriba County, New Mexico, USA, communities with elevated rates of methamphetamine-related morbidity and mortality [20]. The study was designed and implemented in collaboration with community partners including harm reduction agencies and substance use disorder treatment providers. The University of Nevada, Reno Institutional Review Board approved all activities under protocol number 1413390-22.

### Recruitment and data collection

A convenience sample of participants was recruited via street and agency-based outreach. To be included, participants had to be at least 18 years of age and self-report methamphetamine use in the prior 3 months. Participants were recruited via flyers and one-on-one outreach in locations frequented by people who use methamphetamine, including syringe service programs (serving people who are both housed and experiencing homelessness) and encampments of people experiencing homelessness. We also used non-incentivized snowball referral through existing participants.

Data were collected using semi-structured interviews administered by trained research staff, including two authors and other masters-level public health students. Participants were provided the opportunity to be interviewed in English or Spanish, though all interviews were conducted in English. All participants provided written informed consent, received $40 immediately upon providing consent, and were reminded they could end the interview at their discretion. Interviews were recorded and transcribed verbatim. Interviews were conducted at a community-based organization and service provider in New Mexico, and at a University research site in Nevada. Recruitment for qualitative interviews was closed in February 2020 when COVID-19 protocols required shutting down study operations.

A short demographic survey was collected prior to the interview. Then the qualitative interviews asked about participants’ experiences with methamphetamine use and substance use disorder treatment. Then we posed the hypothetical situation of a potential medication for MUD, using the following script: “If there were to be a medication to help you manage your methamphetamine use, how willing would you be to take that? (sort of like medication for opioid use disorder like methadone or buprenorphine, but for methamphetamine)”. We then asked more detailed questions about the goals of taking such a medication (“What would you want this medication to help you with and for how long?”), what would make them willing or unwilling to take the medication, and how they would like to take the medication (route and dosing schedule).

Since this analysis was focused on acceptability of *potential* treatments, the primary questions of interest from the interview were: (1) whether participants would be interested in taking a potential medication for MUD; (2) what effects they would like from such a medication; and (3) what their ideal treatment route and schedule would be (e.g. daily pill, monthly injection). To contextualize participant’s thoughts about treatment preferences within their relationship with methamphetamine use, we also focused on (1) perceived benefits of methamphetamine use and (2) perceived drawbacks of methamphetamine use.

### Analysis

A postdoctoral scholar (author 2), with relevant content experience and expertise in quantitative research, conducted thematic analysis under the guidance of one study PI (author 1), a mixed methods researcher with two decades of qualitative research experience. First, the analyst read every interview in full to become familiar with the content of interviews and created memos documenting initial thoughts. After discussing with PI to achieve consensus, the analyst developed and implemented a set of parent codes (using Atlas.ti qualitative analysis software for data management) to organize interviews by over-arching topics of importance including, but not limited to: medication for MUD; perceived benefits of methamphetamine use; and perceived drawbacks of methamphetamine use. After applying parent codes, quotations ascribed to each parent code were extracted and detailed codes were developed and applied. Regarding medication for MUD, 4 codes were applied: (1) desire to use a potential medication, (2) desired effects of such a treatment, (3) desired route and dosing schedule of such a treatment, and (4) skepticism of the potential efficacy of a medication treatment. The fourth – skepticism – was an unanticipated theme that emerged after initial rounds of coding. In-depth coding of methamphetamine use benefits and drawbacks was applied to full interviews. The analyst then examined medication for MUD code co-occurrence with benefit and drawback codes to explore potential patterns in responses. After drafting an initial write-up of results, the analyst and PI again worked collaboratively to ensure consensus on interpretation and presentation of results. To identify participants quotations, we used an anonymous identifier (e.g., R1, R2, R3) including reported gender and decade of age (e.g., F30 for a woman in her 30s, M20 for a man in his 20s), and the field site (either NV for Nevada or NM for New Mexico). We followed the consolidated criteria for reporting qualitative research (COREQ) checklist for analysis and findings [21].

## Results

Our sample included 21 individuals. One respondent was not asked the questions about medication for MUD and was excluded from the analysis, leaving an analytic sample of 20 (10 from Nevada and 10 from New Mexico). Half identified as women and half as men (Table 1). Median age was 34.5 (range: 23–70), 45% of the sample identified as white and 50% identified as Latinx. Nine people (45%) experienced homelessness in the past 6 months. All participants used methamphetamine, and 13 reported using other drugs in addition to methamphetamine (usually heroin). Thirteen participants had experience taking medication for opioid use disorder (OUD).

**Table 1:** Demographic characteristics (n = 20)

		N	%
Age (median; range)		34.5	23–70
Gender			
Female		10	50
Male		10	50
Latinx		10	50
Race			
Black		2	10
White		9	45
Another race (Hispanic)		6	30
Multiracial		3	15
Homeless in past 6 months		9	45
Completed at least high school education		16	80
Employed full or part time		7	35

First, we describe participants’ perceptions of the benefits and drawbacks of their methamphetamine and other substance use, because these experiences inform how they think about and discuss the potential medication. Then, we describe participants’ opinions about and preferences for a possible medication.

### Benefits of methamphetamine use

When reflecting on the benefits of methamphetamine use, nearly every participant indicated that they liked the energy and positive feelings that methamphetamine provides them:

*Actually, [methamphetamine use] gets me more […] ambitious – you know what I mean? – to want to do something. (R4 F20 NM)*

*But if I have the meth, it really helps me focus. The best part about it is that I feel happy and I can focus. I can get paperwork done. I can write an essay. I can study. Without it, forget about it. Still to this day, forget about it. (R10 F30 NV)*

Among those who also used opioids, participants also said methamphetamine helped them counteract the sedative effects of their opioid use.

*Yeah. I used to use heroin and started using meth a little bit back. I like the way that the energy and the ability to get things done that it gives me that heroin really didn’t kind of little bit of the opposite effect where I feel like I can function better on the day-to-day (R2 M20 NM)*

As described elsewhere [22], participants who were taking medication for OUD also discussed how methamphetamine counteracted the sedation of methadone and suboxone:

*I get tired a lot because of methadone, I’m at a pretty high dose but there’s a reason for that. But [methamphetamine use] keeps me stimulated if I take a hit or two. It’ll keep me up all day for me to – like my energy, just stay going… (R5 F20 NM)*

For some participants, methamphetamine use improved their overall social life and helped them overcome feelings of introversion. Participants experiencing homelessness described not being able to let their guard down around other people, indicating that it was dangerous to do so. Several participants reflected on how methamphetamine allows them to safely navigate social settings:

*I’m a really reclusive kind of person. I don’t like talking to people like in crowds, nothing. I don’t like loud noises, nothing. Meth, it lets me go outside and lets me fucking interact. I don’t worry too much about out it. If I maintain [my methamphetamine use] right and do the right amount, it’s way more sociable. (R11 M20 NV)*

*But now, it’s like I have to be out there with people. [Methamphetamine use] makes it a little bit easier for me to be around so many people all at once because I’d never really like to be exposed but it helps you get our mind off of, you know. It takes your fear of being exposed and being around a lot of people and people paying attention to you. (R20 F40 NV)*

Additionally, several participants discussed how methamphetamine helps them to escape difficult emotions and cope with past traumas:

*I would say it helped me deal with my trauma, I guess. I mean it’s very escaping (R1 F30 NM)*

*R: [Methamphetamine use is] a coping mechanism for me to be able to feel something than nothing….I don’t do well with emotions. Emotions make me extremely vulnerable in my past issues. I’m just not ready to deal with yet, [methamphetamine use] kind of keeps me from having to really deal with those until I’m absolutely ready to… (R19 F40 NV)*

### Drawbacks of methamphetamine use

While nearly all participants reported benefits to their methamphetamine use, reports of drawbacks were not so universal; only around three-quarters of respondents reported drawbacks. When reported, drawbacks included: family problems, paranoia, hallucinations, poor physical health, and withdrawal symptoms.

Several participants discussed how methamphetamine use created complications with their family and loved ones. While some reported that using methamphetamine helped them navigate their parenting duties, others worried that their methamphetamine use may place children at risk through criminalization [e.g. “(my girlfriend) has kids. I worry about that. I don’t want either of us to get into any kind of trouble that might mess things up for the kids…” (R2 M20 NM)]. Others reflected on challenges such as conflict with partners while using methamphetamine.

Participants also described a wide range of mental and emotional drawbacks. Experiences of psychosis, paranoia, getting “freaked out”, and emotional turmoil were common. About a quarter of the participants reported seeing “shadow people”, hallucinations of spirits, monsters, or ghosts after being awake for too long while using methamphetamine:

*I used to see what’s called shadow people and three monsters, like closet monsters. That’s when I know I’ve been up way too long. (R19 F40 NV)*

Experiences with withdrawal symptoms were varied, with some participants reporting no issues while others described excruciatingly painful withdrawal symptoms (e.g. “The come down, when [the methamphetamine is] wearing off, it’s like worse than being sick from heroin.” [R6 M30 NM]). Many participants reported debilitating pain during withdrawal:

*When you start coming off of meth, it feels terrible. Your body feels like broken. Joints hurt. You’re swelling, swelling. You know what I mean? It’s pretty intense. (R21 M30 NM)*

### Acceptability of medication for MUD

We now turn to a discussion of how perceptions about the acceptability of a medication are related to the perceived benefits and drawbacks of methamphetamine use.

Most participants indicated a willingness to try a medication, with many expressing enthusiasm for the potential therapy [e.g. “that would be freaking awesome!” (R4 F20 NM)]. Several were skeptical that such a medication could exist (despite our explanation that one did not *currently* exist) but indicated that they are open to trying anything that might help them reduce their methamphetamine use [e.g. “I feel at this point, I’m willing to try anything” (R1 F30 NM)]. Several indicated that such a medication would benefit their community more broadly.

Five participants indicated they would not be open to a medication. They indicated that they did not view their methamphetamine use as a problem [e.g. “I don’t consider it a problem” (R17 M50 NV)] and each reported zero or only one drawback to their methamphetamine use.

### Medication preferences

#### Functionality and energy—stimulant replacement therapy

As described above, most participants appreciated how methamphetamine provides energy and focus needed to accomplish important life tasks and take care of responsibilities. In alignment with this, respondents indicated that they would prefer a potential medication to provide similar energy and functional benefits:

*I: What would you want that medication to bring? What would it need to do for you in order for it to be a replacement?*

*R: I think as long as to be able to stay energetic and keep that energy coming and being able to do things in life and yeah. Just have that energy, that kick to go with it and not to lose that. (R2 M20 NM)*

As such, many participants articulated the desire for a licit stimulant to replace methamphetamine. Several explicitly identified prescription amphetamines (e.g., Ritalin, Adderall) as an ideal candidate. One participant, when prompted about a potential therapy, indicated that it already exists in the form of such medications:

*I: If there is a similar type of program like a medication-assisted treatment for methamphetamine, what interest in that would you have?*

*R: There kind of is [such a medication]. I mean, [inaudible]. And if I could do it, yeah, I’d do it. Absolutely, I’d take Ritalin or something*. *(R10 F30 NV)*

Part of this connection was related to participants’ understanding of how medication for OUD works, by replacing the use of an illicit opioid (e.g., heroin, fentanyl) with a pharmaceutical alternative (e.g., methadone, buprenorphine). For these respondents, replacing methamphetamine with a prescription amphetamine follows similar logic, by providing benefits without some of the drawbacks.

*I: Given your experience with Suboxone…* *one of the things that [we hope to learn] through this study is some information [about] willingness for methamphetamine type of medications and treatment. What would be your interest or desire or non-desire to utilize something like that?*

*R: That’s an interesting question. I’d be open to maybe Adderall*. *That would be [inaudible] pharmaceutical. It seems to be pretty clean. I tried it before. It’s pretty nice. It helps. It’s just razor -- not razor focus*, *just focus without some of the drawbacks.I’d be open to it. (R13 M20 NV)*

### Preferred treatment schedule

#### Daily dosing—ritual and routine

Most of the participants willing to try a medication indicated a preference for a daily dosing schedule, which would foster a daily routine:

*R: I think the daily [dosing schedule] would be cool. It just gives you something to do and actually gets you on a schedule because if you want to start living in the real world, you have to get up every day to go to work. So, it would start making you get up for something. (R9 F30 NM)*

Several participants discussed how the moment of consuming methamphetamine and the onset of the drug is an important aspect of their use. Some discussed the ritual nature of drug use [e.g. “something I can look forward to, something to replace that part of my day” (R1 F30 NM)], while others reflected on the feeling of onset of the drugs [e.g. “I’d probably be more interested in something where I can feel a sort of like the onset” (R16 M30 NV)].

### Daily dosing—medication for OUD synchronicity

Participants on medication for OUD indicated that it would be helpful if they could receive the daily dosage at the same time and location as their dosage for medication for OUD, which would streamline treatment access and adherence:

*R: [Daily suboxone] seems to work now, and it wouldn’t have to change anything up. It would keep things kind of the same schedule. That could maybe you know, if I was able to go to you know, one trip or not having to see different place. (R2 M20 NM)*

This suggests an opportunity to synchronize medication for MUD and OUD treatment for individuals seeking to reduce both opioid and methamphetamine use.

### Monthly dosing—convenience

Others indicated that they would prefer a monthly injection to a daily dosing schedule, based mostly on convenience. Many noted that remembering to take medication on a regular basis can be challenging for people who use methamphetamine:

*R: For meth [treatment], I don’t see a pill. I see people who do meth just not being able to take the pill because they forget about it or something. They lose them or something. (R11 M20 NV)*

*R: Pills, people forget. If there’s an injection you go like once a month or once every three months or something like that to the doctor’s, and it lasts and if you’re at a level that you know that works, I can see that being possible. (R19 F40 NV)*

Individuals indicating preference for a monthly injection did not express a desire for routine that the participants preferring a daily dosage did. Those responding that they desired a daily schedule largely applied the logic of replacement therapy, with an emphasis on needing a replacement that provides similar functional benefits. Whereas those preferring monthly injections were more likely to reflect on the challenges of adhering to a daily regimen as opposed to reflecting on needing an alternative to provide similar functional benefit.

### Skepticism—medication cannot fix environment

When asked about their interest in a potential medication, a few participants rejected the notion that a medication could, alone, address the root causes of methamphetamine use. One participant responded with heavy sarcasm that, even if it could help him end his methamphetamine use, he would struggle to navigate the same environment, assuming all other things stayed the same:

*R: Okay. That’s great. I’m going to start taking a medication that makes you feel like that that I could get off drugs. Where am I going to go? How am I going to restart again? Okay. This is all going to still be on my mind. (R15 M35 NV)*

## Discussion

We interviewed 20 people who use methamphetamine to explore the perceived acceptability of and preferences for a potential pharmacological treatment for MUD. Most were enthusiastic about the prospect of a replacement-type medicine, with only a few (n = 5) suggesting that they would not be interested because they were not experiencing any significant drawbacks from their methamphetamine use.

Overall, almost every participant said that methamphetamine has important functional benefits, including providing energy, countering the sedation of opioids or medication for OUD, and improving social and emotional wellbeing (including, sometimes, references to self-medicating emotional pain with methamphetamine use). These functional aspects of methamphetamine use have important implications for how respondents thought about a potential medication for MUD and the kinds of treatment outcomes they would consider acceptable. Specifically, respondents talked about desired effects of a medication in ways that suggested a need to mimic the positive and functional aspects of their methamphetamine use, including focus, energy, and routine. This might suggest a need to more thoroughly investigate feasibility and acceptability of abstinence-focused interventions, such as behavioral interventions focused on abstinence rather than reductions in use, and new therapeutics under development, such as vaccines or monoclonal antibodies. Furthermore, it is possible that expanding definitions of acceptable treatment outcomes for MUD beyond abstinence to include reductions of use and/or more regular, routine (rather than chaotic) use could confer clinically important health benefits [18].While not currently approved as an evidence-based treatment modality, stimulant replacement therapy in the form of full or partial agonists (e.g. dextroamphetamine or methylphenidate) is being explored as an approach to mitigating harms associated with stimulant use [23, 24]. Replacement strategies could have a dramatic impact on the increasing rates of death associated with illicit stimulant use in a drug market increasingly characterized by polysubstance use and adulteration with synthetic opioids [25]. Our findings suggest that a replacement-based medication might be acceptable if it can provide some of the same functional benefits.

In contrast to the perceived benefits, which were more universal and commonly described in terms of “energy” and social functioning, the perceived drawbacks of methamphetamine use seemed more individualized and were less clearly linked to ideas about a potential medicine. This was especially true for mental and emotional problems, including psychosis, which manifested in a wide range of ways. While our respondents focused mostly on the psychological impacts, methamphetamine is associated with a host of other health harms. This includes increased risk for cardiovascular illness (hypertension, stroke, cardiomyopathy, heart disease), infectious endocarditis and other bloodborne pathogen transmission from injecting drug use, and increased risk for overdose death (particularly in the context of polysubstance use and co-administration with opioids [26, 27]). The cumulative negative impact of increased exposure to methamphetamine use should not be underestimated, and a pharmaceutical replacement therapy that can eliminate or reduce chaotic use could help mitigate some of the physical (and legal) risks associated with consuming illicit methamphetamine.

Findings about both functional role of methamphetamine and its most common drawbacks reinforce the idea that “good functioning” is a meaningful outcome for evaluating treatment efficacy among people who use methamphetamine. In their examination of relevant clinical endpoints for cocaine use disorder treatment, Carroll et al. operationalized “good functioning” as no cocaine use with no reported legal, employment, psychological, or family problems within the past 28 days [15]. Subsequent studies among people who use methamphetamine suggest that assessing other drug use, legal problems, and psychological concerns may be particularly promising in assessing effects of treatment for MUD [14]. Our findings add to this body of evidence, and highlight social functioning and safety as additional domains. Importantly, a medication that results in cessation of stimulant use (as opposed to stimulant replacement) may leave many individuals without the requisite ability to navigate their environment and responsibilities. Cessation of methamphetamine use through the assistance of a medication would address the health harms associated with the drug, but not the other individual, social, and structural concerns in their lives. This is reflected in the participants’ desire for a medication that mimics the stimulant effect. Interestingly, medication for OUD does not mimic the effects of opioids, in fact, most (methadone and buprenorphine) block the euphoric effects to some extent. The desire for the high or stimulant effect likely reflects the functional aspects of the drug that users value.

Of note in our study and similar to others [28], participants experiencing homelessness relied on methamphetamine to keep them alert and safe around other people, and others relied on methamphetamine to self-medicate underlying mental health concerns. These findings raise concerns regarding some pharmacologic and biological interventions in the clinical trial pipeline, which have the potential to inoculate patients against the stimulant effects of certain drugs with the primary goal of abstinence. Acceptability of these treatments may be limited if they are not accompanied by other therapeutic, social, and economic supports to address underlying concerns.

Other considerations for a potential therapeutic, such as mode and preferences for dosing schedule, were more variable and suggested that treatment regimens must be flexible and tailored to the lived experience of patients. Many participants described how their daily routine is shaped, at least in part, in relation to their methamphetamine use and that an acceptable therapy would provide similar daily structure (e.g., in the form of a daily pill). This was particularly true among those who were also on medication for OUD, since they are already accustomed to a daily dosing schedule that could be synchronized with the new medication. Others, however, perceived daily dosing to be too onerous and wished for a monthly injection that was less likely to be disrupted (like long-acting buprenorphine or naltrexone injections).

Findings should be interpreted in light of the study’s limitations. While qualitative studies are not designed to produce generalizable findings, the findings we report may be subject to influences from the geographic and social context where the study was conducted. Specifically, our data collection sites are located in communities in the Western US with high prevalence of methamphetamine use, and our sample was majority male, white, and had a high prevalence of homelessness. We enrolled people with any reported methamphetamine use and did not screen for MUD, so some people in this sample may not meet the clinical criteria to receive MUD treatment.

## Conclusion

Many participants in our study were excited about the potential for a medication to treat MUD. They applied a logic of substitution therapy when considering such a medication, which reflects the functional role of methamphetamine in their lives and a desire to maintain the stimulant effects while mitigating the harms of illicit methamphetamine. MUD treatment outcomes that focus on patient wellbeing, rather than simply abstinence, should be evaluated, employing a patient-centered perspective to define appropriate clinical endpoints and inform dosing and route of administration could speed adoption and improve uptake of future therapeutics.

## Data Availability

Because of the sensitive nature of the information contained in the transcripts and potential for severe legal and social consequences resulting from broken confidentiality, data will not be made publicly available. Redacted excerpts of the qualitative transcripts used in the current analysis will be made available to qualified researchers subject to review and approval by the appropriate Institutional Review Board(s). Requests can be made to the University of Nevada, Reno Research Integrity Office by calling + 1-775-327-2368.
